# A Study of the Biomechanical Behavior of the Implantation Method of Inverted Shoulder Prosthesis (BIO–RSA) under Different Abduction Movements

**DOI:** 10.3390/bioengineering6010019

**Published:** 2019-02-19

**Authors:** Salah Mebarki, Benaoumeur Aour, Franck Jourdan, Etienne Malachanne, Abdel Hakem Belaghit

**Affiliations:** 1 Laboratory of Applied Biomechanics and Biomaterials (LABAB), Department of Mechanical Engineering, National Polytechnic School of Oran Maurice Audin, Oran 31000, Algeria; ben_aour@yahoo.fr (B.A.); belaghithakim47@gmail.com (A.H.B.); 2 Laboratoire de Mécanique et Génie Civil (LMGC), Université de Montpellier, CNRS, 34090 Montpellier, France; franck.jourdan@umontpellier.fr; 3 C2MA, IMT Mines Ales, Universite de Montpellier, 30100 Ales, France; etienne.malachanne@mines-ales.fr

**Keywords:** shoulder prosthesis, graft, finite elements, abduction, stresses, BIO–RSA

## Abstract

The shoulder is the most mobile joint of the human body, but it is very fragile; several pathologies, and especially muscular degenerations in the elderly, can affect its stability. These are more commonly called rotator cuff fractures. In the case of this type of pathology, the mobility of the shoulder decreases and pain appears. In order to restore mobility and reduce pain, implantation of an inverted shoulder prosthesis is recommended. Unfortunately, over time a notch phenomenon has been observed. In the lower position of the arm, part of the implant comes into contact with the scapula and therefore causes deterioration of the bone. Among the solutions adopted is the lateralized method with bone grafting. However, a main disadvantage of this method concerns the reconstruction of the graft in the case of prosthesis revision. In this context, the aim of the present work was to reconstruct the shoulder joint in 3D in order to obtain a bio-faithful geometry, and then study the behavior of different types of biomaterials that can replace bone grafting. To this end, three arm abduction motions were examined for three individuals. From the results obtained, it appears that grafts in ultra-high molecular weight polyethylene (UHMWPE) exhibit a behavior closer to that of bones.

## 1. Introduction

The shoulder is a suspended joint that is very mobile and very fragile. Thus, it is possible to perform ample movement while ensuring adequate maintenance of its articulation [1]. Shoulder articulation is characterized by weak congruence, which gives it a great amplitude of circumduction, but exposes it to multidirectional instability risks [2].

Many pathological and traumatic factors can disturb this balance. Among these are omarthroses with deficient cuff (with or without subacromial narrowing), comminuted fractures of the humeral head in the elderly and massive ruptures of the cuff bilateral omarthrosis of the shoulder in osteonecrosis [3,4]. The consequences of such pathologies can be dramatic, and involve a lot of pain. According to Lock et al. [5], 20 to 30% of the population is prone to joint pain in the shoulder, and 40% of people over the age of 60 suffer from osteoarthritis on this joint. In the case of appearance of the previously discussed indications, the implantation of an inverted shoulder prosthesis may be recommended to restore mobility and reduce pain.

As an example, in France, each year, around 2700 shoulder prostheses are placed, 30% of which are simple prostheses and 70% of which are total prostheses. Although commonly practiced, postoperative complications persist: Notch, limited movement, instability, etc. [6,7]. The notch problem is the most frequent, according to the Sofcot series [8]. These complications have the effect of prematurely deteriorating the implant (at the level of the cup) and causing pain and other serious consequences for the patient. The appearance of a notch is correlated with deterioration in the clinical outcome that in the medium and long term seems to be worrying. In addition, the polyethylene wear debris generates a granulomatous reaction, responsible for the enlargement of the notch. The latter is one of the complications of the medialization of the rotation center, which is the principle on which the inverted shoulder prosthesis proposed by Paul Grammont is based, used to improve the lever arm of deltoid and to reduce the stresses on the glenoid implant [7].

To treat this problem, several authors have proposed less medialized inverted prostheses than the Grammont prosthesis, the center of rotation of which is closer to the normal shoulder. A case in point is Frankle [7], who proposed a lateralized prosthesis. According to Boileau and Balg [9], prosthetic lateralization undeniably increases the stresses on metaglene, because it yields a significant number of complications. Furthermore, a few have proposed the method BIO–RSA (Bony Increased Offset−Reversed Shoulder Arthroplasty) [10] which is based on the bone lateralization of metaglene using a bone graft taken at the expense of the humeral head. This method gives more interesting clinical results than the method of prosthetic lateralization [9].

To evaluate the effectiveness of shoulder prostheses, several authors developed bio-faithful models of the shoulder, which allowed them to evaluate and analyze the different types of shoulder prostheses by focusing their studies on the fixation mode [11,12,13], the bone grip [14,15,16], the shape and positioning of the prosthesis [17,18], the effect of the prosthesis geometric parameters [19] and the glenoid component stability [20]. Furthermore, a review article published by Berliner et al. [21] discussed the biomechanics of reverse total shoulder arthroplasty, with a focus on elements of implant design and surgical technique that may affect stability, postoperative complications, and functional outcomes. Permeswaran et al. [22], on the other hand, presented cadaveric validation of an FE model (Finite Element Model) developed for studying impingement related to notching in RSA; they found that the numerical model predicted highly focal contact areas due to the sharp edge-on-edge contact present during an impingement event. More recently, Ingrassia et al. [23] presented an evaluation of the reverse shoulder implant design parameters effect on the deltoid muscle forces and on the shoulder range of motion. The obtained results demonstrated that appropriate positioning of the humeral tray can offer significant biomechanical advantages in terms of motion range and abduction force.

Finally, biomaterials chosen for the prosthesis had a great impact on implant performance [24]. These could be divided in several categories. Polymers were chosen for their physical characteristics [25], whereas metal or metal alloys and gold or stainless steel were used despite of allergen risks because they are easy to process and well finished [26,27]. Nowadays, they are progressively being replaced by new materials such as NiTi (Nickel−Titanium) [28] or magnesium and alloys [29] for implants requiring dissolution in biological media.

In this work, we are interested in the BIO–RSA solution (bony-increased offset-reverse shoulder arthroplasty) in the case of revision of the shoulder prosthesis. In order to achieve this goal, a bio-faithful model of the shoulder complex (humerus and scapula) was developed using the 3D segmentation method. Then, we implanted an inverted type prosthesis using the BIO–RSA method. A numerical investigation of the biomechanical behavior of the shoulder complex with prosthesis under different abduction motions of the arm is presented and discussed in detail below.

## 2. 3D Reconstruction and Conception of the Shoulder Prosthesis

**Figure 1:**
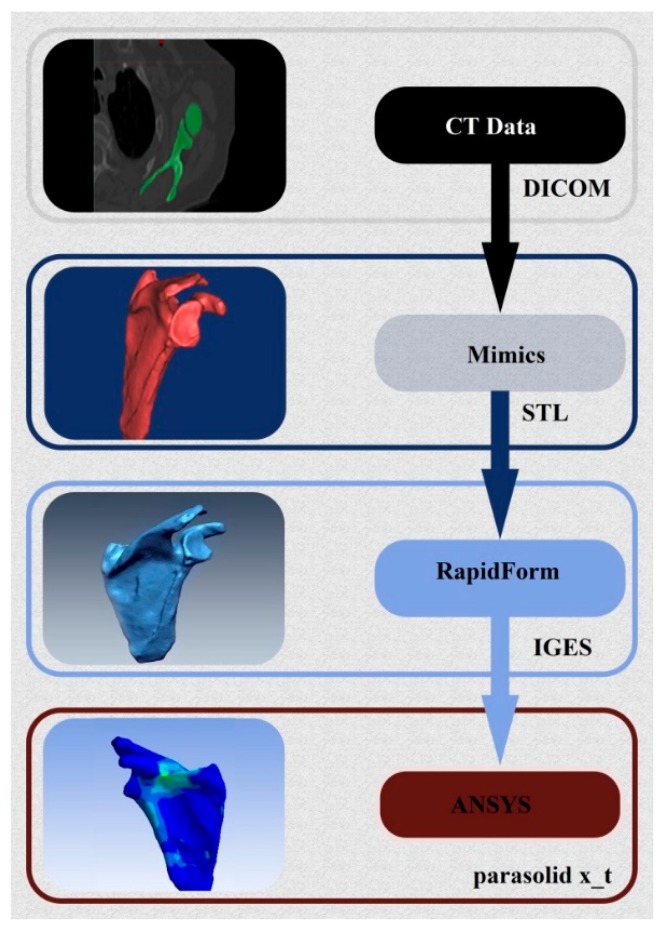
Illustration of the 3D reconstruction procedure of the bone structure of the shoulder.

The model used was constructed by image processing of an MRI file obtained on a subject without a pathology in the shoulder complex, using the technique of 3D reconstruction through several stages. The data conversion method used to develop the finite element model of the shoulder is illustrated in Figure 1. This method is based on tomographic sections taken from radiographs of the shoulder complex performed in a repetitive manner at regular intervals. From these, we can reconstruct a numerical volume model closer to reality. Indeed, the first step is to recover the data from tomographic sections (CT-scan), namely a sequential image file with a 0.5 mm interval, type “DICOM”. X-ray images are transferred to the Mimics image processing software. This anatomical representation software makes it possible to visualize the reconstruction of tomographic sections in 3D. We can see a precise model of the shoulder complex performed with precision in the order of a tenth of a millimeter.

After three-dimensional regeneration of the model using Mimics, the same file is run in the Remesh module to eliminate any debris and correct some of the shape defects (asperities) caused by the segmentation procedure. The resulting model is then exported to an “STL” file. The content of the file is a mesh composed of several triangular facets. However, this mesh cannot be used as is for a finite element calculation. Indeed, segmentation sometimes generates gaps or overlays of elements (discontinuity). In addition, it is performed automatically without any possible control of dimensional parameters of the elements, and does not necessarily respect the constraints necessary for building a mesh of good quality.

It is possible to mesh a surface provided that it is very regular. Indeed, the smoother a surface is, without any alterations and distortions, the better the mesh is. However, the surface generated by Mimics software is not perfect. It is common to find anomalies, most often in the form of folds, where the surface gets wrapped around itself to create a bead. These invisible irregularities disrupt the generation of mesh by creating an infinite number of superimposed elements or elements of almost zero size. For this reason, RapidForm software is used to perform a smoothing operation by filling gaps and rebuilding a regular automatic mesh using NURBS (Non-Uniform Rational Basis Splines) surfaces. The three-dimensional geometric data obtained are converted into the data format of type Parasolid x_t.

**Figure 2:**
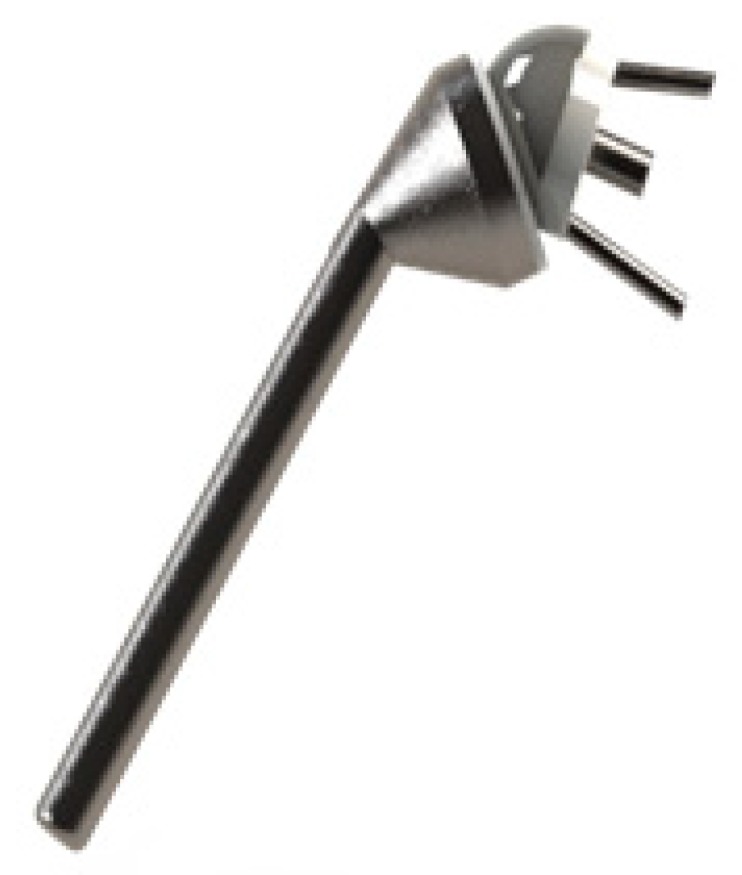
The design of the Delta 3 model of a total inverted shoulder prosthesis.

In this work, the model of Delta3 inverted shoulder prosthesis was chosen for its wide use [30]. Figure 2 illustrates the 3D drawing of this prosthesis using CAD software Solidworks.

**Figure 3:**
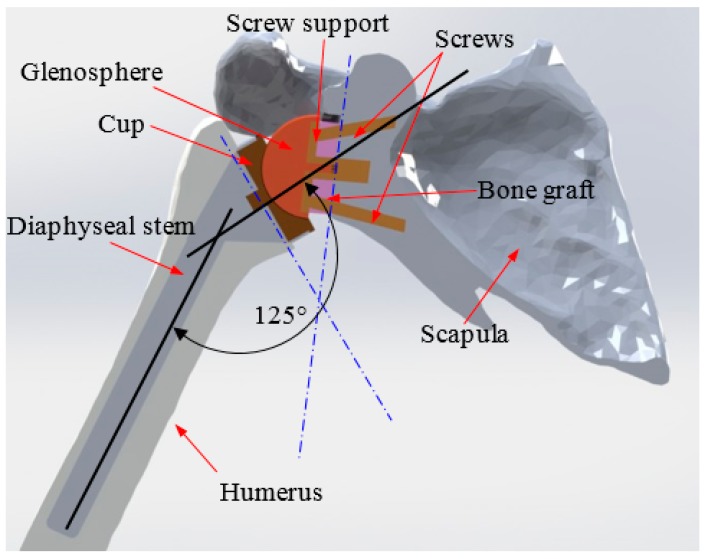
Implantation of the prosthesis into the shoulder joint.

Placement of the prosthesis in accordance with the operative protocol consists of planning the proximal end of the humerus at a cervico-diaphyseal angle of 125°. The glenoid portion should be reduced by a vertical plane in order to position the glenoid base. The humeral part of the prosthesis is inserted in press-fit. On the side of the glenoid, the base is fixed by two screws (Figure 3). This implantation of the prosthesis was carried out in collaboration with experienced surgeons specialized in the placement of this type of prosthesis (Dr. A. Gasmi and his team at the Caduceus Clinic, Oran, Algeria).

## 3. Finite Element Modeling

### 3.1. Mechanical Properties

The different mechanical properties of the prosthesis components and the bones of the shoulder are presented in Table 1.

### 3.2. Loading Conditions

**Figure 4:**
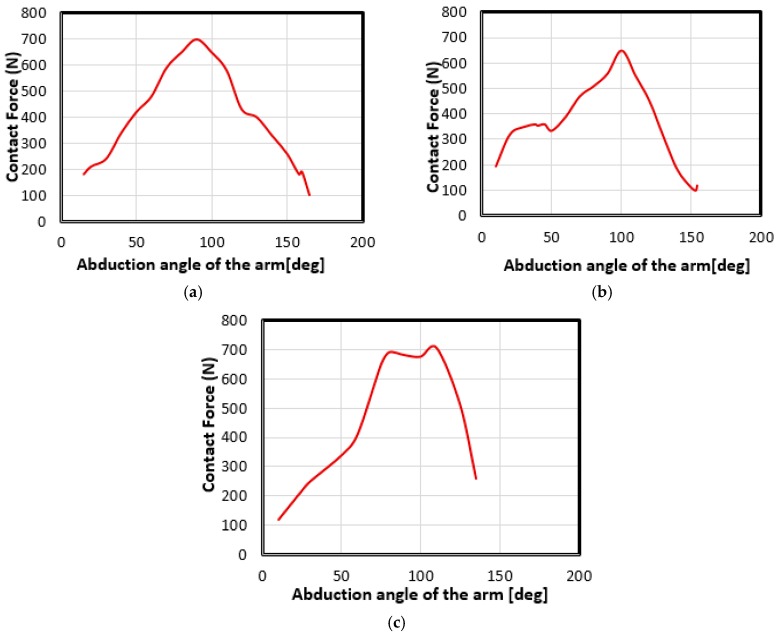
Illustrations of the three models of abduction movement, representing the force applied to the shoulder joint as a function of the abduction angle of the arm in the case of: (**a**) 1st individual, (**b**) 2nd individual and the (**c**) 3rd individual, from [33].

It should be noted that the purpose of this study was to search for biomaterials that could replace bones in the lateralized bone graft method, compare their behaviors and choose the most suitable one. To do this, it was assumed that the compression force undergone by the arm is mainly due to the deltoid muscle, because it allows lifting of the arm. We used three abduction movements of three different individuals. The evolution of the total effort required by the shoulder as a function of the angle of the arm abduction is illustrated by Figure 4 [33]. First, kinematic measurements were preformed using the magnetic movement capture system “Polhemus Liberty”. A sensor was placed on each bone segment (thorax, clavicle, scapula, humerus and forearm) and the system provided their position and orientation over time. Then, a numerical model was developed into the MATLAB software representing the joint glenoid−humeral by a spherical connection and muscles by eleven fibers. An algorithm was developed to calculate the contact reaction force of the glenoid, the strength of the muscles and the contact position of the glenoid, using the kinematic measures of the first part [34].

**Figure 5:**
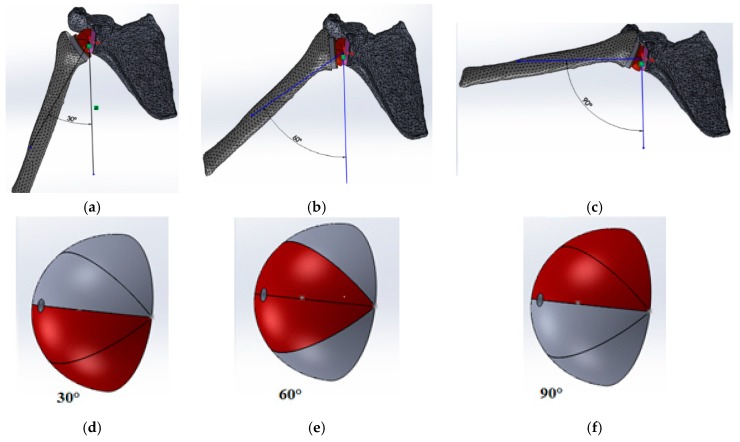
Contact surfaces and zone of applied forces on the glenosphere depending on arm inclination (**a**) 30° position; (**b**) 60° position; (**c**) 90° position; (**d**) applied force for 30°; (**e**) applied force for 60°; (**f**) applied force for 90°.

**Figure 6:**
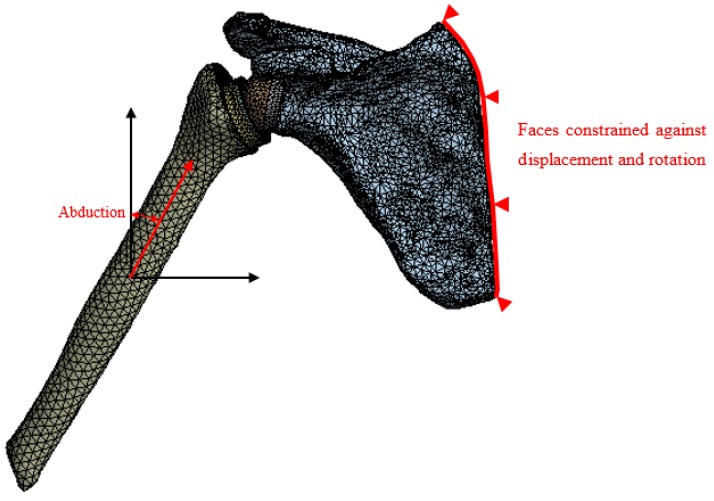
Illustration of the direction of the abduction force as a function of the angle of abduction and the fixed faces of the scapula.

The action of contact of the cup on the glenosphere was modeled by pressure. In fact, the contact force was the compressive force between the humerus head and the glenoid cavity undergone by the arm during abduction movements. The surface of the applied pressure, to get closer to reality, varied depending on the position of the arm by subdividing the glenosphere into four parts as shown in Figure 5. The resulting force of the applied pressure was chosen using the direction shown in Figure 6. The scapula was fixed at its right side (see Figure 6). Simulations were performed in the framework of small strains with linear quasi−static evolutions.

For the glenoid part we defined all contacts as “fully bound” because when the prosthesis is in place, it is assumed to be embedded in the shoulder.

## 4. Results and Discussion

### 4.1. The Mesh Sensitivity Study

**Figure 7:**
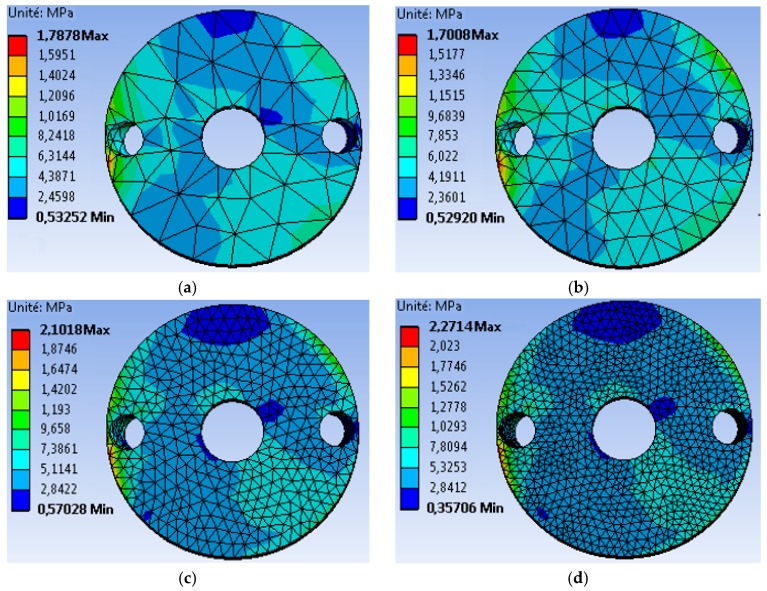
The distribution of Von Mises stresses on the bone graft for different meshes. Mesh (**a**): coarse; Mesh (**b**): medium; Mesh (**c**): fine; Mesh (**d**): very fine.

In order to highlight the effect of the mesh size on the stability and convergence of the results, four different meshes of the graft were examined as illustrated in Figure 7.

- The first mesh (a) of a size of 5 mm was composed of 49,866 tetrahedral elements with 86,186 nodes.
- The second mesh (b) of a medium size (3 mm) was composed of 51,061 tetrahedral elements with 88,109 nodes.
- The third mesh (c) of a fine size (1.5 mm) was composed of 62,567 tetrahedral elements with 105261 nodes.
- The fourth mesh (d) of a very fine size (1 mm) was composed of 93,929 tetrahedral elements with 150,252 nodes.

**Figure 8:**
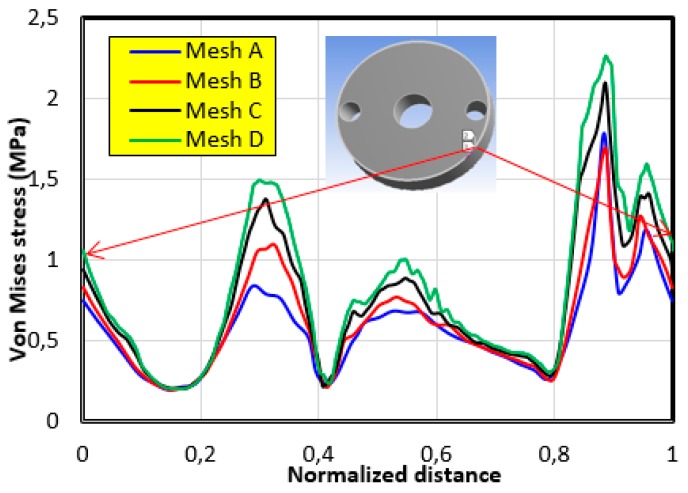
The evolution of the Von Mises stress along the peripheral contour of the bone graft for different meshes.

It should be noted that the same boundary conditions, illustrated in Figure 6, were used with a force of 500 N applied to the contact surface of the glenosphere in the 60° position. From the Von Mises stress distribution, we noticed that there was acceptable agreement between meshes (c) and (d). To confirm these results, we plotted the evolution of the Von Mises stress along the peripheral contour of the graft (Figure 8). This contour was chosen because the maximum value is always localized in this region. It can be seen that there was a rather remarkable difference between the results obtained by meshes (a) and (d), whereas a slight difference was noted between those of (c) and (b). Therefore, in order to reach a better compromise between accuracy and computation time, it is recommended to use a mesh with an element size less than or equal to 1.5 mm.

**Figure 9:**
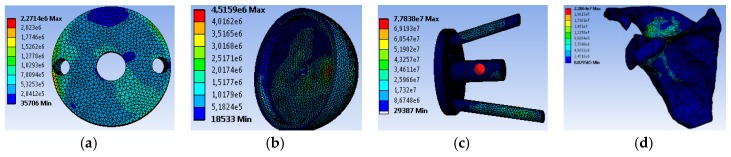
The distribution of Von Mises stresses in the cases of: (**a**) Graft, (**b**) glenosphere, (**c**) support with screws and (**d**) scapula.

Figure 9 shows the Von Mises stress distribution in the cases of: Graft (Figure 9a), glenosphere (Figure 9b), support with screws (Figure 9c) and scapula (Figure 9d), using a very fine mesh. It should be noted that the maximum stress (77.838 MPa) was found at the prosthesis screws, whereas the maximum value at the level of the graft was 2.27 MPa. It should also be noted that the elastic limit for the screws was 830 MPa, whereas that for the bone graft was 135MPa. This satisfies the strength conditions for these components.

After selecting the suitable mesh, we focused on the effects of different abduction movements on the mechanical behavior of the graft by using different biomaterials. The evolution of Von Mises stresses along the graft peripheral contour is highlighted for each individual abduction movement below.

### 4.2. Movement Case of Individual 1

The abduction movement of individual 1 is presented in Figure 4a. Table 2 shows the force applied to the glenosphere as a function of the abduction angle of the arm in abduction motion for the case of individual 1.

**Figure 10:**
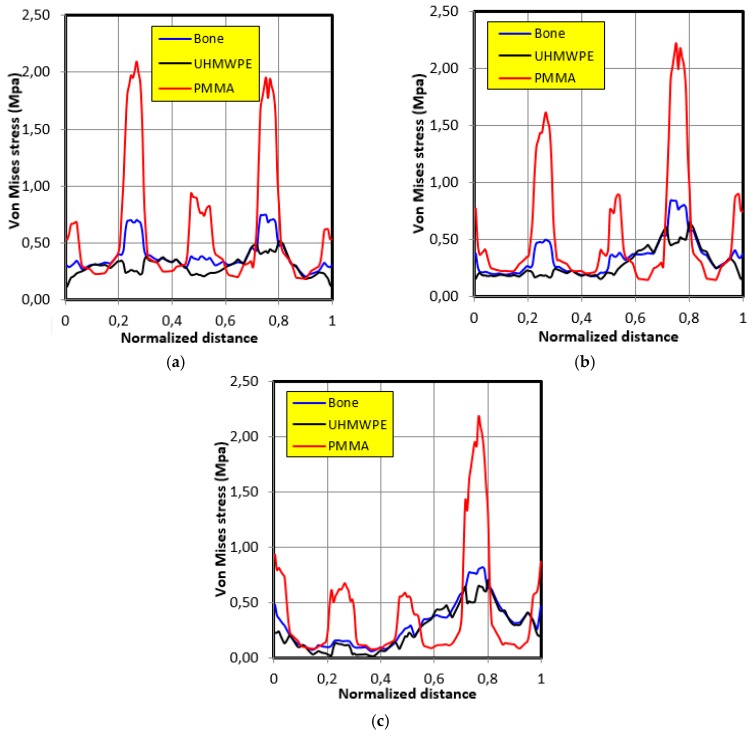
The Von Mises stresses distribution along the graft contour in the case of movement of individual 1. (**a**) 30° position; (**b**) 60° position; (**c**) 90° position.

Figure 10 illustrates the stress distribution along the graft contour for the motion of individual 1. It can be noted that the stress variation in ultra-high molecular weight polyethylene (UHMWPE) was closer than that of bone grafting. It is also important to note that the maximum stress in the cases of bone and polyethylene UHMWPE were 0.8 MPa and 0.7 MPa, respectively, whereas in the case of polymethyl methacrylate (PMMA), this was 2.18 MPa.

### 4.3. Movement Case of Individual 2

The abduction movement of individual 2 is presented in Figure 4b. Table 3 shows the force applied to the glenosphere as a function of the abduction angle of the arm in abduction motion for the case of individual 2.

**Figure 11:**
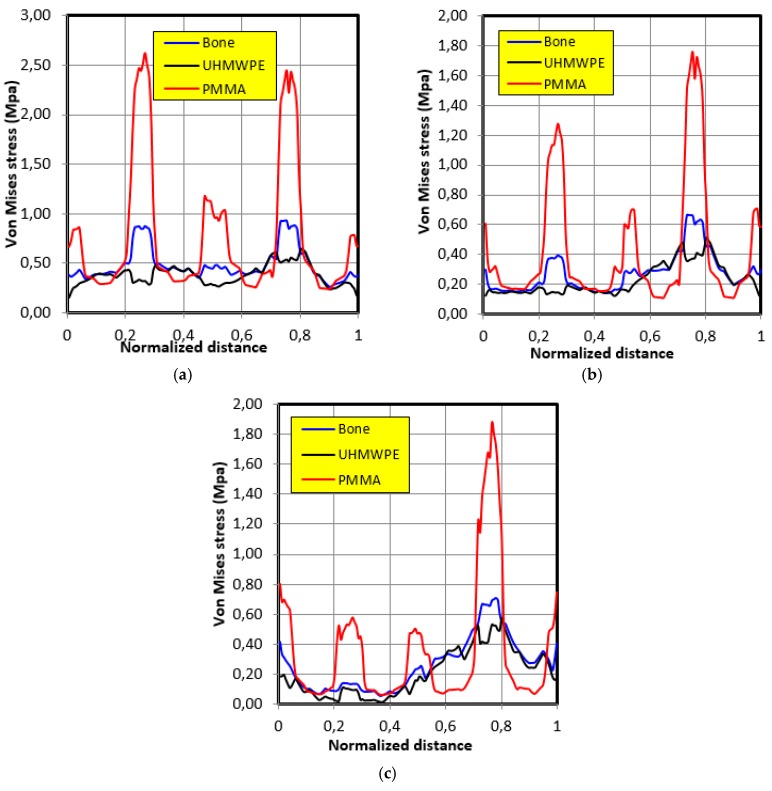
The Von Mises stresses distribution along the graft contour in the case of movement of individual 2. (**a**) 30° position; (**b**) 60° position; (**c**) 90° position.

The distribution of stresses along the contour of the graft in the case of movement of individual 2 is shown in Figure 11. The same tendencies of variation of stresses as those of the movement of individual 1 were noticed in the second individual; that is, the behavior closest to the bone was that of polyethylene UHMWPE. In the second individual, the maximum stresses were as follows: 0.85 MPa in the case of bone, 0.6 MPa in the case of polyethylene UHMWPE, and 2.6 MPa in the case of PMMA.

### 4.4. Movement Case of Individual 3

The abduction movement of individual 3 is presented in Figure 4c. Table 4 shows the force applied to the glenosphere as a function of the abduction angle of the arm in abduction motion in the case of individual 3.

**Figure 12:**
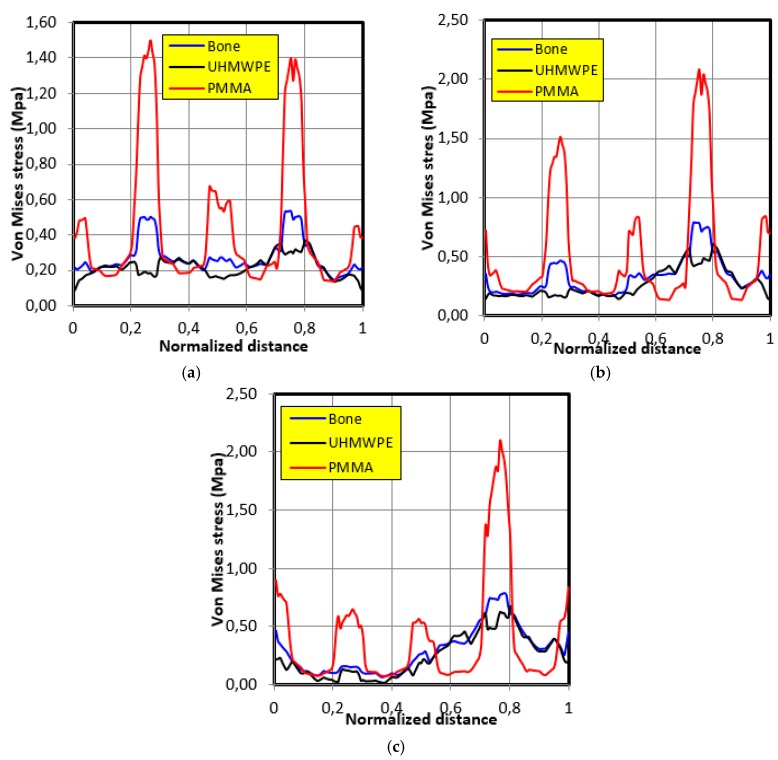
The Von Mises stresses distribution along the graft contour in the case of movement of individual 3. (**a**) 30° position; (**b**) 60° position; (**c**) 90° position.

Figure 12 shows the distribution of Von Mises stresses along the contour of the graft in the case of the third individual’s movement. The same tendencies of stresses variation as those of the first individual movement were noted; that is, the behavior closest to the bone was that of polyethylene UHMWPE. It was also noted that the maximum stress in the case of bone was 0.75 MPa, whereas in the case of polyethylene UHMWPE it was 0.6 MPa, and in the case of polymethyl methacrylate PMMA it was 2.1 MPa.

The highest maximum stresses for the PMMA and bone grafts were obtained in the case of individual 2, when the contact surface of the glenosphere was in the 30° position, whereas for the UHMWPE graft, the highest maximum stress was obtained in the case of individual 1, when the contact surface of the glenosphere was in the 90° position.

These numerical results deserve a verification with experimental ones. We did not make any experimental measurements, and unfortunately the lack of experimental data in the literature did not allow us to compare numerical and experimental stress results.

It is important to note that the differences between UHMWPE and PMMA were very large; however, the obtained results for UHMWPE were very similar to those obtained for bone. Indeed, stresses peaks were located on either side of the holes. This is normal because holes are geometric singularities that lead to mechanical singularities. The difference in stresses intensity can only be attributed to the properties of the materials. The Young modulus and the Poisson ratio between PMMA, bone and UHMWPE are significantly different. It is the combination of these material parameters that created their differences.

Nevertheless, we could notice that the yield strengths for UHMWPE and PMMA were around 25 MPa and 70 MPa, respectively, which are far from the maximum computed stresses.

## 5. Conclusion

The main objective of this study was to propose a solution to the problem of bone graft regeneration in the case of revision of the implanted inverted shoulder prosthesis using the BIO–RSA method. In order to achieve this goal, we started by designing the prosthesis using Solidworks software. In the second phase, we reconstructed the anatomical model of the shoulder in 3D using Mimics and RapidForm software. ANSYS Workbench software was used to conduct a finite element analysis of different efforts applied to the prosthetic shoulder with different biomaterials of the graft. Emphasis was placed on the abduction motion of the arm because it is this gesture that most solicits the articulation of the shoulder. This movement was studied in three precise positions: 30°, 60° and 90° for three individuals.

From the results obtained, we can draw the following conclusions:

- To obtain reliable results, we must use bio-faithful geometric models with a fine mesh.
- The graft of ultra-high molecular weight polyethylene (UHMWPE) has a closer mechanical response to that of bone compared to poly methacrylate (PMMA). So, grafts made of UHMWPE minimize stress and better protect the prosthesis from the risk of fatigue failure.
- The maximum stress on the graft varies significantly depending on the properties of the graft, the position of the contact surface and the individual.

Finally, from a clinical point of view, this study shows that it is better to use a graft made of UHMWPE than PMMA to minimize the stresses created close to holes. Furthermore, in order to properly examine the consequences of lateralization in RSA, studies on multiple variables such as the extent of lateralization, basal plate orientation and screw orientation could be considered. It should also be noted that the possibility of performing 3D printing to produce specific grafts for each patient is a very interesting surgical guideline.

## Figures and Tables

**Table 1:** Properties of bone and prosthetic materials in shoulder arthroplasty [31,32].

Elements	Materials	Young Modulus E(MPa)	Poisson Coefficient ν	Density (g/mm^3^)
**Scapula**	Cortical bone	8000	0.3	1.3 × 10^−3^
**Screws and Screws Holder**	Titanium	110,000	0.33	4.5 × 10^−3^
**Glenosphere**	Stainless steel	230,000	0.3	8.01 × 10^−3^
**Graft**	PMMA	2000	0.22	-
	UHMWPE	500	0.4	-
	Bone	450	0.3	-

**Table 2:** Applied force to the glenosphere as a function of the elevation angle of the humerus in the case of individual 1.

**Contact Force (N)**	**280**	**480**	**710**
**Elevation of the Humerus (Degrees)**	30	60	90

**Table 3:** The applied force to the glenosphere as a function of the elevation angle of the humerus in the case of individual 2.

**Contact Force (N)**	**350**	**390**	**560**
**Elevation of the Humerus (Degrees)**	30	60	90

**Table 4:** The applied force to the glenosphere as a function of the elevation angle of the humerus in the case of individual 3.

**Contact Force (N)**	**250**	**410**	**680**
**Elevation of the Humerus (Degrees)**	30	60	90

## References

[1] Astier V., Arnoux P.J., Thollon L., Mouret F., Brunet C. Finite element simulation of humeral intramedullary nailing: Case of torsion loading. Presented at 2nd European Hyperworks Technology Conference Strasbourg.

[2] Walch G., Edwards T.B., Boulahia A., Nové-Josserand L., Neyton L., Szabo I. (2005). Arthroscopic tenotomy of the long head of the biceps in the treatment of rotator cuff tears: Clinical and radiographic results of 307 cases. J. Shoulder Elbow Surg..

[3] Lévigne C., Lacroix P. (2010). Jérome Shoulder arthroplasty in 2010 anatomical or reversed, prosthesis? Indications and contra-indications. Rev. Rhum. Monogr..

[4] Boussakri H., Alassaf I., Hammoudi S., Elidrissi M., Shimi M., Elibrahimi A., Elmrini A., Dumez J.F. (2015). Total bilateral reverse shoulder prosthesis: About two cases. Pan Afr. Med. J..

[5] Lock C., Allgar V., Jones K., Marples G., Chandler C., Dawson P. (1999). Prevalence of back, neck and shoulder problems in the inner city. Physiother. Res. Int..

[6] Boileau P. (2016). Complications and revision of reverse total shoulder arthroplasty. Orthop. Traumatol. Surg. Res..

[7] Frankle M., Siegal S., Pupello D., Saleem A., Mighell M., Vasey M. (2005). The Reverse Shoulder Prosthesis for glenohumeral arthritis associated with severe rotator cuff deficiency. A minimum two-year follow-up study of sixty patients. J. Bone Jt. Surg. Am..

[8] Kolmodin J., Davidson I.U., Jun B.J., Sodhi N., Subhas N., Patterson T.E., Li Z.M., Iannotti J.P., Ricchetti E.T. (2018). Scapular Notching After Reverse Total Shoulder Arthroplasty. J. Bone Jt. Surg..

[9] Boileau P., Balg F. (2008). The reverse shoulder prosthesis: Biomechanical principles, concept and evolution Prothèses D’épaule. État Actuel.

[10] Kirzner N., Paul E., Moaveni A. (2018). Reverse shoulder arthroplasty vs BIO-RSA: Clinical and radiographic outcomes at short term follow-up. J. Orthop. Surg. Res..

[11] Ahir S.P., Walker P.S., Squire-Taylor C.J., Blunn G.W., Bayley J.I. (2004). Analysis of glenoid fixation for a reversed anatomy fixed-fulcrum shoulder replacement. J. Biomech..

[12] Couteau B., Mansat P., Darmana R., Mansat M., Egan J. (2000). Morphological and mechanical analysis of the glenoid by the geometric reconstruction using computed tomography. Clin. Biomech..

[13] Terrier A., Buchler P., Farron A. (2005). Bone-cement interface of the glenoid component: Stress analysis for varying cement thickness. Clin. Biomech..

[14] Lacroix D., Murphy L.A., Prendergast P.J. (2000). Three-dimensional finite element analysis of glenoid replacement prosthesis; a comparison of keeled and pegged anchorage systems. J. Biomech. Eng..

[15] Murphy L.A., Prendergast P.J., Resch H. (2001). Structural analysis of an offset-keel design glenoid component compared with a center-keel design. J. Shoulder Elbow Surg..

[16] Clavert P., Zerah M., Krier J., Mille P., Kempf J.F., Kahn J.L. (2006). Finite element analysis of the strain distribution in the humeral head tubercles during abduction: Comparison of young and osteoporotic bone. Surg. Radiol. Anat..

[17] Büchler P., Farron A. (2004). Benefits of an anatomical reconstruction of the humeral head during shoulder arthroplasty, a finite element analysis. J. Biomech..

[18] Terrier A., Farron A. Biomechanical analysis of reversed shoulder prosthesis: Benefit of the inferior position of the glenoid base plate. Proceedings of the 52nd Annual Meeting of the Orthopaedic Research Society.

[19] Langohr G.D., Willing R., Medley J.B., Athwal G.S., Johnson J.A. (2015). Contact mechanics of reverse total shoulder arthroplasty during abduction: The effect of neck-shaft angle, humeral cup depth, and glenosphere diameter. J. Shoulder Elbow Surg..

[20] Chae S.-W., Lee H., Kim S.M., Lee J., Han S.-H., Kim S.-Y. (2016). Primary Stability of Inferior Tilt Fixation of the Glenoid Component in Reverse Total Shoulder Arthroplasty: A Finite Element Study. J. Orthop. Res..

[21] Berliner J.L., Regalado-Magdos A., Ma C.B., Feeley B.T. (2015). biomechanics of reverse total shoulder arthroplasty. J. Shoulder Elbow Surg..

[22] Permeswaran V.N., Goetz J.E., Rudert M.J., Hettrich C.M., Anderson D.D. (2016). Cadaveric validation of a finite element modeling approach for studying scapular notching in reverse shoulder arthroplasty. J. Biomech..

[23] Ingrassia T., Nalbone L., Nigrelli V., Ricotta V., Pisciotta D. (2018). Biomechanical analysis of the humeral tray positioning in reverse shoulder arthroplasty design. Int. J. Interact. Des. Manuf..

[24] Ansari F., Lee T., Malito L., Martin A., Gunther S.B., Harmsen S., Norris T.R., Ries M., van Citters D., Pruitt L. (2016). Analysis of severely fractured glenoid components: Clinical consequences of biomechanics, design, and materials selection on implant performance. J. Shoulder Elbow Surg..

[25] Saini M., Singh Y., Arora P., Arora V., Jain K. (2015). Implant biomaterials: A comprehensive review. World J. Clin. Cases.

[26] Ko J.-W., Nicholson T.A., Hoffler C.E., Williams G., Getz C. (2017). Metal Allergy as a Cause of Implant Failure in Shoulder Arthroplasty. Orthopedics.

[27] Ghadikolaei A.D., Vahdati M. (2014). Experimental study on the effect of finishing parameters on surface roughness in magneto-rheological abrasive flow finishing process. Proc. Inst. Mech. Eng. Part B J. Eng. Manuf..

[28] Ibrahim H., Jahadakbar A., Dehghan A., Moghaddam N.S., Amerinatanzi A., Elahinia M. (2018). In Vitro Corrosion Assessment of Additively Manufactured Porous NiTi Structures for Bone Fixation Applications. Metals.

[29] Amerinatanzi A., Mehrabi R., Ibrahim H., Dehghan A., Moghaddam N.S., Elahinia M. (2018). Predicting the Biodegradation of Magnesium Alloy Implants: Modeling, Parameter Identification, and Validation. Bioengineering.

[30] Clark J.C., Ritchie J., Frederick, Song S., Kissenberth M.J., Tolan S.J., Hart N.D., Hawkins R.J. (2012). Complication rates, dislocation, pain, and postoperative range of motion after reverse shoulder arthroplasty in patients with and without repair of the subscapularis. J. Shoulder Elbow Surg.

[31] Astier V., Thollon L., Arnoux P.J., Mouret F., Brunet C. (2008). Development of a finite element model of the shoulder: Application during a side impact. Int. J. Crashworth..

[32] Quental C., Folgado J., Fernandes P.R., Monteiro J. (2015). Computational analysis of polyethylene wear in anatomical and reverse shoulder prostheses. Med. Biol. Eng. Comput..

[33] Isabelle P., Silvio R., Cyntia D., Alexandre T. (2009). Analyse Cinématique et Biomécanique de l’épaule lors d’activités de la vie Quotidienne. EPFL Scientific Publications. http://ibi.epfl.ch/files/content/sites/sti/files/shared/sgm/masterprojects/Poster_Pretre.pdf.

[34] Sarshari E. (2018). A Closed-Loop EMG-Assisted Shoulder Model. Es Sciences. Ph.D. Thesis.

